# Head and Neck Squamous Cell Carcinoma with Distant Metastasis: A Systematic Review and Meta-Analysis

**DOI:** 10.3390/cancers16223887

**Published:** 2024-11-20

**Authors:** Antonio Daloiso, Leonardo Franz, Tiziana Mondello, Matteo Tisato, Michael Negrisolo, Paolo Zanatta, Cosimo de Filippis, Laura Astolfi, Gino Marioni

**Affiliations:** 1 Otolaryngology Section, Department of Neuroscience DNS, University of Padova, 35131 Padova, Italy; antonio.daloiso@aopd.veneto.it (A.D.); matteo.tisato@aopd.veneto.it (M.T.); michael.negrisolo@studenti.unipd.it (M.N.); 2 Phoniatrics and Audiology Unit, Department of Neuroscience DNS, University of Padova, 31100 Treviso, Italy; leonardo.franz@unipd.it (L.F.); cosimo.defilippis@unipd.it (C.d.F.); 3 Department of Anesthesiology and Critical Care, Treviso Hospital, 31100 Treviso, Italy; paolo.zanatta1@aulss2.veneto.it; 4 Bioacoustics Research Laboratory, Department of Neuroscience DNS, University of Padova, 35131 Padova, Italy; laura.astolfi@unipd.it

**Keywords:** squamous cell carcinoma, head and neck, distant metastasis, systematic review, meta-analysis

## Abstract

Background/Objectives: Distant metastasis (DM), though uncommon at initial presentation, significantly worsens the prognosis of head and neck squamous cell carcinomas (HNSCCs). This review aimed to investigate the occurrence rates, patterns, and implications of HNSCC DM. Methods: A systematic search was performed in Scopus, PubMed and Web of Science. Results: Out of 7576 identified titles, 35 studies were included, encompassing 28,193 patients. The pooled rate of DM was 10.01%, with significant heterogeneity existing among the studies (I2: 94.13%). The most common metastatic sites were the lungs, bones, and brain (58%, 15%, 4%, respectively). Treatment modalities varied: overall, 20.4% of patients received radiotherapy alone, 7% underwent chemotherapy, and 4.5% received surgical metastasectomies. Combined treatments accounted for 18.3% of patients. However, 41.3% of patients received no treatment for DM. The median overall survival (OS) after DM diagnosis was 10.1 months. Studies highlighted a 36.3% two-year survival rate for patients with oligo-metastases, compared to the 7.4% rate for those with multiple metastases. At the time of DM diagnosis, half of the studied population presented with locoregional failure. Conclusions: Advanced imaging techniques and emerging systemic therapies offer hope for improved DM detection and treatment. However, continuous research is essential to develop therapeutic strategies that can enhance survival and improve the quality of life for patients with DM.

## Simple Summary

Distant metastasis (DM), though uncommon at initial presentation, significantly worsens the prognosis of head and neck squamous cell carcinomas (HNSCCs). This systematic review and meta-analysis aimed to investigate the occurrence rates, patterns, and implications of DM. Out of 7576 identified titles, 35 studies were included, encompassing 28,193 HNSCC patients. The pooled rate of DM was 10.01%, with significant heterogeneity existing among the studies. The most common metastatic sites were the lungs, bones, and brain. Treatment modalities varied: overall, 20.4% of patients received radiotherapy alone, 7% underwent chemotherapy, and 4.5% received surgical metastasectomies. Combined treatments for DM accounted for 18.3% of patients. However, 41.3% of patients received no treatment. The median overall survival after DM diagnosis was 10.1 months. The strength of the available evidence is currently too weak to drive robust clinical recommendations. Advanced imaging techniques and emerging systemic therapies offer hope for the improved detection and treatment of DM.

## 1. Introduction

Head and neck squamous cell carcinoma (HNSCC) ranks as the sixth most common cancer worldwide. Annually, approximately 850,000 cases are diagnosed, leading to significant morbidity and mortality [1]. The diagnosis and treatment of HNSCCs improved significantly over the last decades, but long-term overall survival (OS) did not experience similar progress, especially in advanced cases [2]. Lymph node metastasis remains the most important unfavorable prognostic factor, affecting both regional control and survival [3]. In this landscape, distant metastases (DM) can make the prognosis even worse, occurring in less than 5% of cases at presentation and affecting 3 to 52% of cases throughout the course of the disease, a rate exceeding 40–50% in autopsy studies [4,5].

Hematogenous metastatic spread primarily targets the lungs, bones, and liver, dictating the need for appropriate staging at HNSCC diagnosis, vigilant surveillance, and treatment [5,6,7,8,9,10]. The advent of advanced imaging techniques, notably (18)F-fluorodeoxyglucose positron emission tomography ((18)F-FDG PET), has improved the detection of DM, offering a hint of hope for the development of an early therapeutic approach [11,12,13]. Furthermore, recent developments in systemic therapies, including immunotherapy, and local ablative techniques have been deemed promising for the treatment of DM, with the potential to prolong patient survival and improve quality of life [14]. However, despite advances in the treatment of HNSCCs, the understanding of the clinical behavior and metastatic patterns remains incomplete, with a mean OS around 10.1 months in distant metastatic HNSCCs [15].

This systematic review and meta-analysis aimed to provide an overview of the occurrence, patterns, and clinical implications of DM in HNSCC, as well as to report on the treatment strategies currently available.

## 2. Materials and Methods

### 2.1. Protocol Registration

The protocol of this systematic review was registered on PROSPERO, an international database of prospectively registered systematic reviews in health and social care (Center for Reviews and Dissemination, University of York, York, UK), in January 2024 (registry number CRD42024499146).

### 2.2. Search Strategy

A systematic literature review was conducted according to the recommendations of the Preferred Reporting Items for Systematic Reviews and Meta-Analyses (PRISMA) [16]. The electronic databases Scopus, PubMed, and Web of Science were searched from database inception until 15 January 2024. The search strategy combined various medical subject headings and text words for DM, head and neck and squamous cell carcinoma, and the upper aerodigestive tract (distant AND “metast*” AND “squamous” AND (carcinoma OR tumor) AND (head and neck OR upper aerodigestive tract)). The reference lists of all the included articles were thoroughly screened to find other relevant articles. References were exported to Zotero bibliography manager (v6.0.10, Center for History and New Media, George Mason University, Fairfax, VA, USA). After the removal of duplicates, two reviewers (A.D. and T.M.) independently screened all titles and abstracts and then evaluated the full texts of the eligible articles based on the inclusion criteria. Any disagreement between the reviewers involved in the literature search was resolved through discussion with all authors to reach a consensus.

### 2.3. Selection Criteria

Studies were deemed eligible when the following inclusion criteria were met: (i) a histopathologically confirmed diagnosis of HNSCC; (ii) clearly reported data on DM. Exclusion criteria were as follows: (i) a lack of relevant data; (ii) a series of head and neck primary skin or salivary gland carcinomas; (iii) a non-original studies (i.e., reviews, recommendations, editorials, conference papers, clinical challenges and book chapters); (iv) animal model studies; (v) non-English language studies; (vi) case reports and case series with less than 50 patients; (vii) studies with overlapping patient cohorts or studies with patient populations with exclusively metastatic disease; (viii) studies which considered national database analysis. Articles were also excluded when they did not have a clear follow-up duration or did not report the incidence of DM and other relevant clinical data.

### 2.4. Data Extraction and Quality Assessment

The data extracted were collected in an electronic database. The data included the first author, year of publication, country of origin, study design, enrollment period, sample size, number of metastatic patients, primary site and metastatic site, treatment strategy, and survival data.

The quality of the studies eligible for inclusion was categorized as poor, fair, and good, in agreement with the National Institutes of Health quality assessment tool for Observational Cohorts and Cross-Sectional Studies [17]. Two reviewers (A.D. and T.M.) independently evaluated the papers, and any disagreement was solved by discussion.

### 2.5. Statistical Analysis

A meta-analysis was conducted to compare the DM rate across the studies considered. The size’s effect, evaluated for the purpose of the meta-analysis, was the proportion of cases with DM throughout the sample of each study, considering the relative standard error. Considering the possible differences in study methods, patients’ characteristics, and practice patterns, we decided to use a random effects model, which accounted for between-study variations in terms of effect size (DM rate). The mean of the normal distribution of the logarithm of the effect size across studies represented the estimated overall average value, and the variance in such a normal distribution represented the between-study variability. Forest plots were produced to display the mean and 95% confidence interval (C.I.) for the DM rate pooled in our meta-analysis.

Heterogeneity was assessed using the I2 statistic, which described the percentage of total variation across studies present due to heterogeneity rather than chance [18]. A I2 value of 0% was assumed to indicate no heterogeneity, while values around 25%, 50%, and 75% could suggest low, moderate, and high heterogeneity, respectively [18]. Heterogeneity was investigated by performing subgroup analysis and meta regression.

The presence of publication bias was explored using the funnel plot and the Egger test based on regression for small-study effects. 

The meta suite of Stata 16 (Stata Corp., College Station, TX, USA) was used for all statistical analyses. 

## 3. Results

### 3.1. Search Results and Quality Assessment

**Figure 1:**
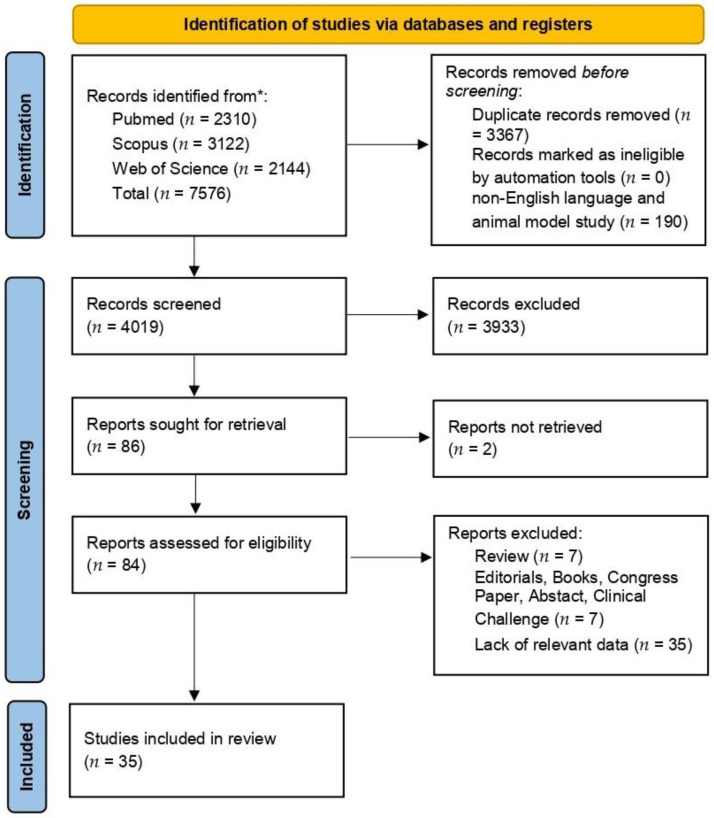
PRISMA diagram summarizing electronic database search and inclusion/exclusion process of the review. Legend * date of last search 15 January 2024.

A total of 7576 titles were collected from our literature search. After the removal of duplicates and the exclusion of 3933 records due to the degrees of coherence with the inclusion/exclusion criteria, 84 articles relevant to the topic were examined. No records were unavailable for retrieval. Finally, 35 articles were included in the review. A detailed flowchart of the search process is shown in Figure 1. 

Working in accordance with the National Institutes of Health quality assessment tool for Observational Cohorts and Cross-Sectional Studies [17], 11 studies (31.4%) were deemed to be of good quality, 20 (57.1%) to be fair, and 4 (11.4%) to be poor. The latter assessment was due to the lack of reporting information on the series’ features.

### 3.2. Series Description

All studies included in the qualitative analysis had an observational retrospective design [9,19,20,21,22,23,24,25,26,27,28,29,30,31,32,33,34,35,36,37,38,39,40,41,42,43,44,45,46,47,48,49,50,51,52]. Studies were published between 1977 and 2023, encompassing patients treated from 1948 to 2019. A total of 28193 patients were included from the data collected in the investigations under review. In general, the patients reported in these studies had an average age ranging between 48 and 64 years (median age from 55 to 65 years). Data on patients’ demographics, the study design, and the prevalence of metastatic patients in each included article are reported in Table 1.

The site of the primary carcinoma varied among the 35 studies mentioned above (Table 2). The oropharynx was the primary site in 29 studies (82.8%), the oral cavity in 24 (68.5%, among which one focused on tongue SCC), the hypopharynx in 17 (48.5%), the larynx in 16 (45.7%), the paranasal sinuses in 5 (14.3%), and the nasopharynx in 5 (14.3%), with cancer of unknown primary (CUP) seen in 3 (8.5%) cases. The frequency of multiple metastatic patients among all patients with DM was 3.3% (756/2360) (Table 2). Some research groups evaluated UAT altogether [9,24,26,27,28,29,30,33,34,38,40,41,42,43,52], whereas others focused on fewer primary sites, for instance the oral cavity/oropharynx [19,20,21,22,23,31,32,35,37,39,44,45,46,47,48,50,51], larynx/hypopharynx [25], or the tongue alone [49]. HPV status was analyzed in 7 studies [22,31,32,37,45,46,51].

Patients included in these investigations were variably distributed between the different pathological stages of the disease based on tumor TNM, as shown in Table 2. Overall, 8 investigations reported the TNM of the whole cohort [21,29,34,36,39,40,46], whereas more than half of them (22/35) highlighted the TNM classification of patients who subsequently developed DMs [9,20,22,23,25,26,27,28,30,32,33,35,36,37,38,41,43,44,45,47,48,49,50]. Some clinical research groups did not specify the tumor stage [19,24,31,50,52]. In 20 studies [9,20,23,25,26,27,28,30,32,33,36,37,38,41,43,44,45,47,48,50], DMs occurred in more than half of patients with advanced T and N classifications, while only 3 studies showed the opposite trend [22,35,49].

Comprehensively, almost 10% (2775/28193) of the HNSCC patients included in the considered series developed DM after an average period ranging between 12 and 23 months from initial primary tumor diagnosis (Table 3; also shown in the pooled analysis in Section 3.3). Some clinical research groups evaluated the site of metastasis based on the whole DM group, establishing whether two or more synchronous metastatic sites were present; others extrapolated the proportion based on the total number of DM, without a clear distinction between oligo- and poly-metastasis (Table 3). The most common site of metastases was the lung, followed, in descending order, by the bones, brain, skin and liver (this is also shown in the pooled analysis in Section 3.3). Moreover, locoregional failure occurred at the time of DM diagnosis in half of the population under study. 

The treatment modalities used are reported in Table 3. Overall, 25 articles mainly focused on the treatment of the primary tumor [9,19,20,21,23,25,28,29,30,35,36,38,39,40,41,42,43,44,45,47,48,49,50,51,52], 6 also described the treatment of DM [22,24,26,32,37,46], whereas the remaining 4 did not describe any therapy [27,31,33,34]. The 6 articles focusing on DM treatment accounted for 601 patients. Radiotherapy alone was the approach most used to control DM (20.4%, 123/601). This encompassed brachytherapy, intensity-modulated radiotherapy, and stereotactic radiosurgery, as shown in Table 3. Overall, 7% (43/601) of patients received chemotherapy, whereas 4.5% (27/601) underwent surgical metastasectomy, such as pulmonary lobectomy [22,24,36,38,43]. However, combined treatments were the second most favored treatment (18.3%, 110/601), with concomitant chemoradiotherapy used in 12% of cases, and surgery preceded by induction chemotherapy or followed by adjuvant radio- or chemotherapy used in others (6.4%). It is worth mentioning that 41.3% of patients did not undergo any kind of treatment for DM and data on DM therapy were unavailable in 50 patients [22,24,26].

Patients’ survival was analyzed in 26 investigations (Table 3). Six studies reported the OS, disease-specific survival (DSS), and disease-free survival (DFS) of all patients [19,23,25,29,31,35,36,37,38,39,41,44,45,46,51,52]; conversely, the remaining investigations focused on the DFS based on the DM status (DMFS) and on the OS after the diagnosis of DM. Working in terms of oligo- or poly-metastasis, the OS was evaluated in 3 studies as 36.3% and 7.4% for an average period of 2 years, respectively [22,38,41]. In another 2 investigations, OS was based on local-recurrence control (LRC) or failure (LCF), which were 31.8% and 1.4%, respectively, for an average period of 3.5 years [35,38]. The median OS after DM diagnosis was 14.6 months (range 5 to 34 months) in 6 studies, while DMFS was 67% at 1 year and 29% at 10 years [21,29,30,34,36,38,39,40,42,43,44,45,46,52]. The time from DM diagnosis to death was calculated by Calhoun et al., who worked for an average of 3.4 months [26].

### 3.3. Pooled Analysis

**Figure 2:**
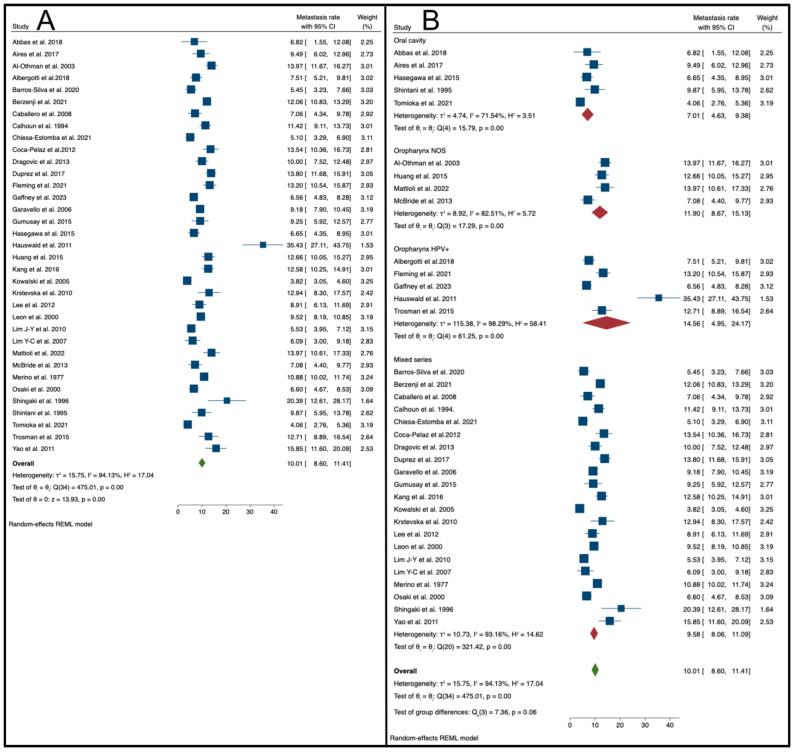
(**A**) Forest plot reporting the mean DM rate (with 95% CI) across all the studies included [9,19,20,21,22,23,24,25,26,27,28,29,30,31,32,33,34,35,36,37,38,39,40,41,42,43,44,45,46,47,48,49,50,51,52]. (**B**) Subgroup analysis: forest plots summarizing the mean DM rate (95% CI) stratified by primary carcinoma sites (oral cavity, oropharynx NOS, oropharynx [HPV+], and mixed series) [9,19,20,21,22,23,24,25,26,27,28,29,30,31,32,33,34,35,36,37,38,39,40,41,42,43,44,45,46,47,48,49,50,51,52].

Considering the 35 studies that provided enough clinical data, the overall pooled metastasis rate was 10.01% (95% C.I.: 8.60–11.41%, as also shown in Figure 2), even if large and significant heterogeneity values were found (I2: 94.13%, *p* < 0.01).

**Figure 3:**
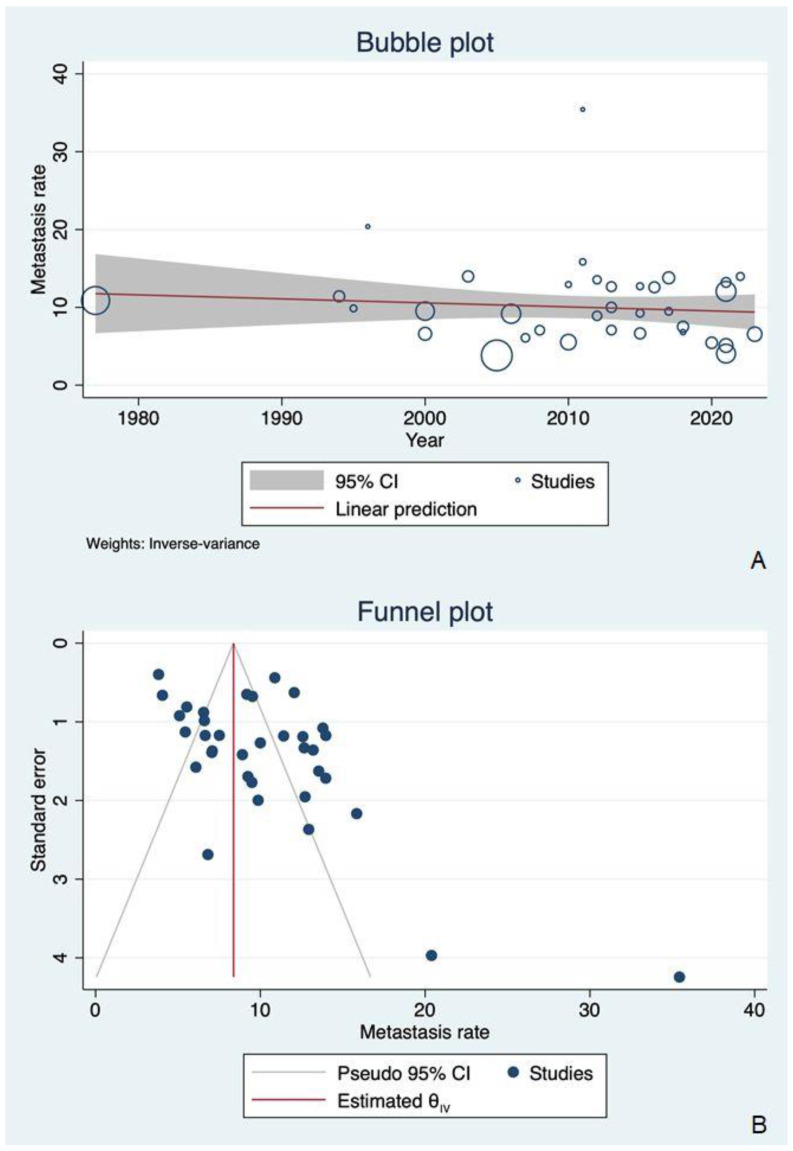
(**A**) A bubble plot summarizing the distribution of DM rates by publication year: no significant change in DM rate over time was visible in the included articles (coefficient = −0.0514, R^2^ = 0.00, *p* = 0.484). (**B**) A funnel plot showing a slightly asymmetrical distribution of DM rates, suggesting a possible publication bias. This could reasonably be due to the small study effect.

A subgroup analysis, performed by primary tumor site, showed that studies based on HPV-positive oropharyngeal cancers had a higher pooled metastasis rate (14.56%; 95% C.I.: 4.95–24.17%; I2: 98.29%, *p* < 0.01). The lowest rate was observed in series of oral cavity carcinomas (7.01%; 95% C.I.: 4.60–9.38%; I2: 71.54%, *p* < 0.01). Intermediate rate values were observed in series of HNSCCs from mixed and not otherwise classified sites (9.58%; 95% C.I.: 8.06–11.09%; I2: 93.16%, *p* < 0.01), and in series from oropharynx tissue with an unspecified HPV status (11.90%; 95% C.I.: 8.67–15.13%; I2: 82.51%, *p* < 0.01). Details are given in Figure 3. In addition, considering the oral cavity subgroup as a reference, the meta regression by primary tumor site confirmed a significantly increased risk of metastasis in studies dealing with HPV-positive oropharyngeal carcinomas (coefficient: 5.54; 95% CI: 0.16 to 10.92; *p* = 0.044; information is also given in Table 4).

The meta regression model based on publication year ruled out any significant change in DM rate over time (coefficient= −0.0514, R^2^ = 0.00, *p* = 0.484; this is also shown in Table 4 and Figure 3).

Looking at the geographic region in which each population was based, the meta regression model revealed a significantly lower risk of metastasis in studies from Asia (Coefficient: −3.47; 95% CI: −6.73 to −0.22; *p* = 0.037) when compared to those based in Europe (this can also be seen in Table 4).

**Figure 4:**
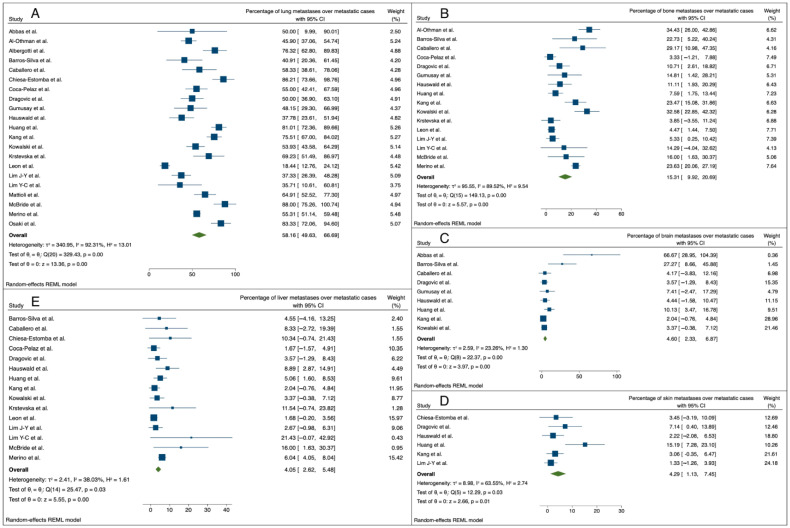
Forest plots reporting the mean involvement rate (with 95% CI) of each metastasis site over the metastatic cases: (**A**) lung metastasis rate [9,19,21,22,23,25,27,28,29,34,36,37,38,39,40,42,43,44,45,46,47]; (**B**) bone metastasis rate [9,21,23,25,28,29,34,36,37,38,39,40,42,43,44,46]; (**C**) brain metastasis rate [19,23,25,29,34,36,37,38,39]; (**D**) skin metastasis rate [27,29,36,37,38,43]; (**E**) liver metastasis rate [9,23,25,27,28,29,36,37,38,39,40,42,43,44,46].

Considering the distribution of DM sites, the pooled analysis identified lung as the organ most frequently involved, accounting for the 58.16% of metastatic cases (95% C.I.: 49.63–66.69%). Bone was the second most frequently involved site, being affected in 15.31% of cases (95% C.I.: 9.92–20.69), while brain, skin, and liver hosted distant metastases in around 4% of metastatic cases (4.60%, 95% C.I.: 2.33–6.87; 4.29%, 95% C.I.: 1.13–7.45; and 4.05%, 95% C.I.: 2.62–5.48, respectively; this is also shown in Figure 4).

**Figure 5:**
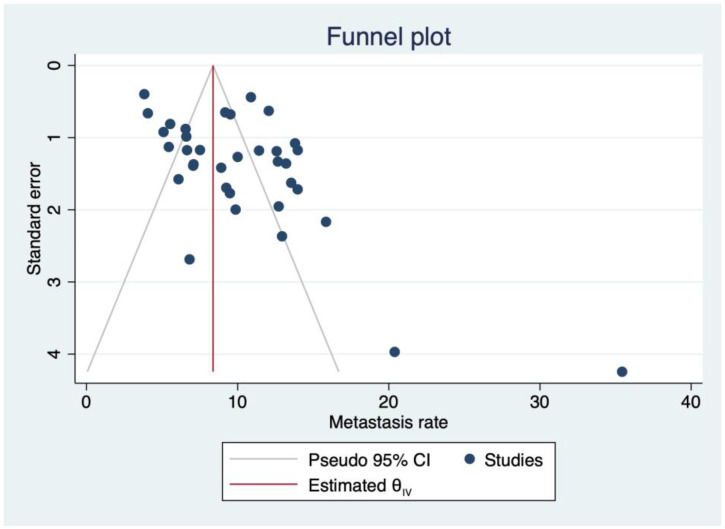
A funnel plot showing a slightly asymmetrical distribution of DM rates, suggesting a possible publication bias. This could reasonably be due to the small study effect.

The relatively skewed distribution of studies within the funnel plot, as shown in Figure 5, and the evidence of a significantly small study effect in the regression-based Egger test (*p* < 0.001) suggested the possible presence of publication biases.

## 4. Discussion

DM was defined as the spread of the tumor to other organ systems. The HNSCC spread can be of two types, namely, (1) non-lymphatic metastases (hematogenous spread), more commonly affecting the lung, bone, liver, and skin, or (2) lymphatic metastases beyond regional lymph nodes, with the frequent involvement of mediastinal, abdominal, and axillary nodes [53,54]. Distant metastasis can manifest in two distinct ways: synchronously, where metastases are already present at the initial diagnosis of the primary tumor, indicating an advanced stage of disease from the outset; or metachronously, where metastases develop after an interval following the initial treatment or diagnosis of the primary tumor, signifying the progression or recurrence of the disease over time [55].

### 4.1. Detection and Incidence of Distant Metastases

In this systematic review, the overall incidence of DM was 10.01%. This was determined based on the analysis of studies from 1977 to 2023. The most common sites of DM were the lungs, bones, skin, brain, and liver. Multiple metastases were observed on average in 3.3% (756/2360) of the patients (Table 2). Studies highlight the variability in the incidence of DM based on factors like the tumor’s anatomical location, HPV status, and regional lymph node involvement. Innovations such as PET/CT and MRI, or the more recently developed PET-MRI, have significantly improved the sensitivity and specificity towards the detection of DM [55]. A recent meta-analysis of 23 published studies showed the excellent results of using PET/CT to detect DM in HNSCCs at 12 months after locoregional treatment [56]. This integration of functional and anatomical imaging heralds a new era in the precise identification and assessment of DM, facilitating more tailored and effective treatment approaches. Additionally, emerging techniques such as liquid biopsies and the analysis of circulating tumor DNA (ctDNA) are aimed at transforming the early detection and monitoring of metastatic disease [57].

### 4.2. Predictive and Prognostic Factors

Predictive markers of DM in HNSCCs incorporate a wide spectrum of clinical and pathological indicators, notably advanced T and N stages, primary tumor site, the presence of extracapsular extension (ENE), locoregional failure, and HPV positivity in oropharyngeal SCCs (OPSCCs) [11,30,33,42,52,58,59,60].

Tumor size is a crucial determinant of the surgical approach, with more advanced carcinomas requiring more extensive resections and causing challenges in the maintenance of clear surgical margins [19,20,26,33,49,52]. An advanced T-stage increases the risk of neck lymph node involvement, locoregional recurrence, and ultimately, poor prognosis. However, the relationship between tumor size and DM remains a subject of debate [33,39,43,61]. Tumor thickness has recently been recognized among the risk factors for a worse prognosis in patients with oral SCC, alongside a higher incidence of lymph node metastasis and locoregional recurrence [62]. However, this has not yet been established for DM patients [63].

The primary tumor site has a significant impact on the incidence of DM development. Several studies suggested that the hypopharynx is the site with the highest probability of subsequent DM [27,28,30,33,38,40,42,52,53,64]. Kang et al. found that the risk of DM was significantly higher in patients with oral and hypopharynx carcinoma compared to those with oropharyngeal disease. Specifically, the hazard ratio (HR) for the development of DM in oral cancer was 1.97. For hypopharyngeal carcinoma, the hazard ratio was notably higher (3.03) [38]. Similarly, Leon et al. reported that the hypo- and naso-pharyngeal carcinoma exhibited the highest frequency of DM; oropharynx and supraglottic carcinoma showed an intermediate frequency of DM [42]. Although the exact reasons for the high propensity for DM occurrence among hypopharynx and larynx carcinomas are not well understood, the rich lymphovascular supply of these regions might play a role in this [65].

However, in our meta-analysis, it was not feasible to make comparisons with all other head and neck sites, as most of the studies included often referred to mixed series rather than individual ones [9,24,25,26,27,28,29,33,34,36,38,40,41,42,43,52]. This did not allow us to create any specific categories for hypo- and naso-pharyngeal carcinomas in the subgroup analysis. Instead, specific pooled DM rates could be obtained by subgroup analysis for HPV-positive cancers of the oropharynx and oral cavity carcinomas. The fact that the pooled metastasis rate was higher in the series of HPV-positive oropharyngeal cancers compared to the oral cavity and the mixed series is consistent with the well-known peculiar metastasis pattern typical of such virus-induced tumors [51,66]. This can also be seen in Section 4.3.

Cervical lymph node metastasis is recognized as a significant predictor of DM, especially when there is the involvement of a larger number of lymph nodes, positive contralateral lymph nodes, and levels IV–V involvement [9,19,20,21,23,24,25,26,27,28,29,30,33,34,35,40,41,42,43,44,50]. In this setting, ENE is known to worsen the prognosis of HNSCC patients, thus prompting adjuvant chemotherapy and radiotherapy treatment, according to the currently accepted approaches [25,67,68]. Nonetheless, ENE’s association with DM presents conflicting evidence; only a few studies found a significant impact [25,30,33,35,42], while others did not show an actual correlation, which was probably due to limited series [27,39,41,43].

Locoregional recurrence stands out as a pivotal risk factor in terms of DM development [19,20,21,29,33,34,35,40,41,42,43,45]. There are a relevant number of patients with locoregionally advanced disease without DM, and cancer-related morbidity and mortality are mainly due to locoregional disease [9,20,21,29,30,33,35,38,41,42,43,50,52]. In a solid retrospective study, patients with extensive nodal disease and those with locoregional failure experienced significantly higher DM rates, with locoregional failure being the most significant predictive factor. Moreover, regional disease recurrence was reported as a more relevant predictor of DM compared to primary site failure [29]. Merino et al. analyzed more than 5000 patients treated for HNSCCs from 1948 to 1973, finding a 16.7% incidence of DM in those with failure above the clavicles, compared to a 7.9% incidence in patients with locoregional control [9]. In this systematic review, the comprehensive frequency of DM associated with locoregional recurrence was 50%.

In general, the prognosis of patients with HNSCCs with DM is poor, characterized by considerable variability. This is influenced by metastatic burden, sites of metastasis, and the overall health status. However, a subset of patients with oligo-metastatic disease may achieve long-term survival, particularly when aggressive local therapies are feasible, highlighting the importance of comprehensive disease treatment. There is significant heterogeneity in survival among DM patients, i.e., those with fewer metastatic foci seem to experience better OS rates compared to patients with poly-metastasis [22,24]. This aligns with the concept of oligo-metastasis, introduced in 1995 by Hellman and Weichselbaum [69], suggesting that metastasis should not be seen as a binary state but rather as a spectrum of metastatic disease. Also, in HNSCC, comparing oligo- and poly-metastasis states seemed to predict survival, with significantly better outcomes observed in oligo-metastatic cases [22,38,41]. However, the exact number of metastatic foci and locations defining oligo-metastasis remains unclear. Similar results were observed in our review. Specifically, oligo-metastasis was associated with a significantly higher overall survival (OS) rate of 36.3% at a 2-year average follow-up, compared to the OS of 7.4% seen for poly-metastasis [22,38,41].

### 4.3. HPV-Related Carcinomas and Distant Metastasis

The discussion around HPV-associated OPSCCs reflected a nuanced understanding of their behavior. Despite the generally better outcomes associated with HPV-positive OPSCCs, up to 11% of patients displaying them experienced DM, underscoring their serious prognosis [37]. There is increasing evidence showing that HPV-positive tumors might be associated with delayed metastases, which might also be related to the long survival in these patients [37]. The literature addressing whether HPV-positive patients demonstrate unique patterns of DM progression remains equivocal [66]. Recent studies suggested that the treatment outcomes for recurrent or metastatic HPV-positive OPSCCs patients were notably better compared to those seen in HPV-negative cases [70,71]. These findings further distinguished the unique metastatic patterns observed in HPV-positive OPSCCs, which are characterized by metachronous, diffuse poly-metastasis [51]. In the investigations by Sayed et al., patients with HPV-positive OPSCCs had a 2-year survival rate after DM diagnosis of 45.3% compared to HPV-negative patients, who had a rate of 11.3% [72]. McBride et al. reported that HPV-positive patients with metastatic OPSCCs had 1- and 2-year survival rates of 72.0% and 40.8%, respectively [46]. Mainly examining mainly patients that were non-surgically treated, Huang et al. [37] found a 2-year survival rate of 11% and 4% after DM diagnosis for HPV-positive and HPV-negative OPSCC patients, respectively. In a small cohort of 37 OPSCCs patients, Trosman et al. [51] reported better outcomes in HPV-positive patients than HPV-negative ones, with a median OS values after DM diagnosis of 25.6 months and 11.1 months, respectively.

### 4.4. Treatment Strategies

The standard-of-care management of DM in HNSCCs has traditionally been palliative, with systemic therapies offering limited prospects in terms of a cure. However, the landscape is evolving, with aggressive interventions for oligo-metastatic disease and the advent of targeted and immunotherapies that show promise in altering prognostic outcomes [22,73]. Addressing DM in HNSCCs entails a multifaceted strategy that includes systemic therapies, targeted treatments, and, in selected cases, surgical interventions or localized radiation therapy [41].

With regard to systemic treatment in metastatic or recurrent SCC, the EXTREME regimen, consisting of cetuximab combined with a platinum-based drug (cisplatin or carboplatin) and 5-fluorouracil (5-FU), could be a first-line treatment [74]. This regimen has been shown to prolong median survival by approximately 2.7 months and progression-free survival by 2.3 months compared to chemotherapy alone in recurrent or metastatic HNSCCs [74]. The notable advantages of the EXTREME regimen not only included improved survival but also the better quality of life due to a significant improvement in pain control, in eating, and in speaking [74]. As reported, an alternative to the EXTREME regimen is the TPEx protocol, which uses taxane instead of 5-FU and shows promising results in phase II trials [75].

Currently, there is a growing consensus around the notion that an aggressive approach to the treatment of oligo-metastatic disease can significantly enhance patient survival. Such treatments are increasingly tailored to the individual patient’s disease profile, with the aim of optimizing efficacy while minimizing side effects [76]. The local control of metastatic sites in HNSCCs can significantly improve outcomes, particularly in patients with oligo-metastatic disease [24,77]. Surgical resection and stereotactic body radiation therapy are two primary methods of limited metastatic treatment. Surgical resection is commonly considered for lung metastases in oligo-metastatic HNSCC cases [22,46,78,79]. In terms of surgical outcomes, there was a median 5-year overall survival rate of about 29% for patients undergoing lung metastasectomy [80]. Stereotactic body radiation therapy for lung metastases has shown promising control rates and survival outcomes, being comparable to the outcomes of surgical resection in selected cases [22,81].

A multidisciplinary approach is essential for the treatment of metastatic HNSCCs, involving a combination of systemic therapy, local control measures, and supportive care tailored to the general condition of the patient and specific characteristics of the disease [65]. This approach aims to maximize quality of life and prolong survival while managing the complex dynamics of metastatic disease [65].

The advent of artificial intelligence (AI) and machine learning has the potential to revolutionize oncology with the aim of improving diagnostic precision, predicting patient outcomes, and customizing treatment strategies with unparalleled precision [82]. By sifting through extensive datasets, AI algorithms can detect patterns and correlations that elude human detection, potentially unveiling new biomarkers and/or therapeutic targets for DM in HNSCCs [83]. This could pave the way for AI-enhanced diagnostic tools and decision-support systems, optimizing treatment effectiveness while reducing adverse effects [83]. In our study, data on treatment modalities were obtained based on the pooled analysis of the articles included. Given the wide evaluation timespan, therapeutic approaches could have varied significantly over the 40-year period. It is also crucial to consider previous treatment modalities for the primary tumor that might have influenced the decision about the appropriate approach to metastasis.

### 4.5. Limitations of the Study

The limitations of this study are primarily related to the fact that all included articles were observational and retrospective. This design inherently carries biases and limits the ability to establish definitive causal relationships. The included investigations displayed considerable differences in terms of patient demographics, study design, primary tumor sites, and diagnostic and treatment approaches. This heterogeneity could affect the consistency of results and may complicate the interpretation and generalizability of pooled findings. Additionally, some studies also lacked comprehensive reporting of clinical data, detracting from the depth of analysis and interpretation of the results. A limitation of this analysis is the variability in imaging techniques across studies, as well as the absence of detailed imaging data, which prevented direct comparison. Older studies lacking access to advanced imaging methods, such as PET/CT or PET/MRI, may potentially have underestimated the incidence of DM. However, as disclosed by the pooled analysis, no significant modification of the DM rate was reported over the considered time span, despite a dramatic evolution of diagnostic tools.

The diverse surgical techniques and adjuvant therapies reported across the studies also show challenges in conclusively assessing the effectiveness of specific treatments. Furthermore, the lack of detailed data on pathogenic factors (e.g., alcohol or tobacco exposure) or histopathologic characteristics (e.g., histological variants) may have hindered between-study comparisons. Lastly, the available data and the inclusion criteria of the studies in our review did not allow for consistent comparisons with control cohorts, which could have provided additional context.

## 5. Conclusions

In HNSCCs, the epidemiology, biological behavior, and treatment of DM can largely vary depending on the primary tumor site. Managing HNSCCs requires careful attention to DM risk factors, especially in patients with high-risk profiles, including high-stage tumors, advanced nodal disease, or HPV-positive OPSCCs. As a result, the implementation of more intensive treatments of primary tumors in high-risk subjects, along with tailored surveillance strategies, may lead to a reduction in the DM occurrence. According to the current literature, the management of HNSCCs with DM is evolving. Despite the variety of reported DM treatment approaches, they can be classified into the following domains: (i) systemic treatments (e.g., the EXTREME regimen [74] or TPEx protocol [75]); (ii) localized treatments for oligo-metastatic disease, including surgical resection [41] or stereotactic radiotherapy [81]; (iii) multidisciplinary approaches integrating systemic, local, and supportive therapies to improve quality of life and survival. However, the current evidence is not robust enough to support strong clinical recommendations, a task which requires prospective multi-center studies. To develop stronger evidence-based clinical recommendations, future research should be based on homogeneous samples, preferably in multi-center, prospective, and controlled settings. Personalized approaches that are based on AI algorithms and trained on large datasets should also be considered.

## Figures and Tables

**Table 1:** Characteristics of the studies included in the systematic review.

Author	Year	Country	Enrollment Period	Age (years)	Sex (No.; M/F)	Considered Patients (No.)	Metastasis Incidence (%)	Primary SCC Site
Abbas et al. [19]	2018	Pakistan	2006–2013	48.3 * ± 12 (22–72)	61/27	88	6.8	O
Aires et al. [20]	2017	Brazil	2009–2015	59.9 * ± 10.9 (33–91)	210/64	274	9.6	O
Al-Othman et al. [21]	2003	USA	1983–1997	NA	693/180	873	14	OP
Albergotti et al. [22]	2018	USA	1980–2015	PolyM: 59 * ± 11; OligoM: 59 * ± 7	NA	506	7.5	OP HPV+
Barros-Silva et al. [23]	2020	Brazil	2000–2014	<65 yrs: 214 cases (53%) ≥65 yrs: 9190 cases (47%)	290/114	404	5.4	O; OP
Berzenji et al. [24]	2021	Netherlands	2006–2013	64.3 * ± 9.5	239/85	2687	12.4	O; OP; HP; L; NP; Sinus; CUP
Caballero et al. [25]	2008	Spain	1998–2004	NA	321/19	340	7.1	HP; L
Calhoun et al. [26]	1994	USA	1975–1987	59.4 ^§^ (29–81)	NA	727	11.4	O; OP; HP; L
Chiesa-Estomba et al. [27]	2021	Spain	2016–2020	63.91 * ± 11.7 (25–84)	471/98	569	5.1	O; OP; HP; L; NP
Coca-Pelaz et al. [28]	2012	Spain	1999–2006	60.2 * (33–86)	431/12	443	13.5	O; OP; HP; L
Dragovic et al. [29]	2013	USA	1995–2007	57.5 ^§^ (23–92)	450/110	560	10	O; OP; HP; L
Duprez et al. [30]	2017	Belgium	1992–2015	61 ^§^ (31–95)	919/103	1022	13.8	O; OP; HP; L; CUP
Fleming et al. [31]	2021	USA	2001–2018	60 ^§^ (42–82)	NA	621	13.2	OP HPV+
Gaffney et al. [32]	2023	UK	2011–2019	61.5 ^§^ (37–84)	NA	793	6.6	OP HPV+
Garavello et al. [33]	2006	Italy	1981–1998	62 * (33–82)	1855/117	1972	9.2	O; OP; HP; L
Gumusay et al. [34]	2015	Turkey	2000–2010	55 ^§^ (18–84)	228/64	292	9.2	O; OP; HP; L; NP
Hasegawa et al. [35]	2015	Japan	2001–2014	65.9 * ± 13.5 (23–97)	271/180	451	6.6	O
Hauswald et al. [36]	2011	Germany	1992–2005	55 ^§^ (32–79)	110/17	127	35.4	OP; HP
Huang et al. [37]	2013	Canada	2000–2010	HPV+: 57 ^§^ (27–92) HPV−: 65 ^§^ (33–88)	482/142	624	12.7	OP
Kang et al. [38]	2016	South Korea	2006–2011	60 ^§^ (20–88)	641/138	779	12.6	O; OP; HP; L; NP; Sinuses
Kowalski et al. [39]	2005	Brazil	1954–1997	<65 yrs: 1734 cases; ≥65 yrs: 593 cases	1982/624	2327	3.8	O; OP
Krstevska et al. [40]	2010	North Macedonia	1999–2004	57 ^§^ (34–79)	172/29	201	12.9	O; OP; HP; L
Lee et al. [41]	2012	South Korea	2005–2009	59 * (20–87)	334/70	404	8.9	O; OP; HP; L
León et al. [42]	2000	Spain	1984–1996	NA	1157/87 (LRC pts)	1880	9.5	O; OP; HP; L; NP
Lim J-Y et al. [43]	2010	South Korea	1991–2007	60 * (20–87)	559/72	795	7	O; OP; HP; L
Lim Y-C et al. [44]	2007	South Korea	1992–2004	56 * (20–79)	185/45	230	21	O; OP
Mattioli et al. [45]	2022	Italy	2009–2019	63 * (55–70)	312/96	408	14	OP
McBride et al. [46]	2013	USA	2002–2010	58 ^§^ (43–80)	NA	353	7.1	OP
Merino et al. [9]	1977	USA	1948–1973	NA	NA	5019	10.8	O; OP; HP; L; Sinus
Osaki et al. [47]	2000	Japan	1970–1998	NA	NA	636	6.6	O; OP; Sinus
Shingaki et al. [48]	1996	Japan	NA	53 * (26–83)	68/35	103	20.4	O; OP; Sinus
Shintani et al. [49]	1995	Japan	1976–1990	53.8 * (25–81)	129/94	223	9.9	O (Tongue)
Tomioka et al. [50]	2021	Japan	2008–2017	64.4 * (24–87)	573/354	887	4	O
Trosman et al. [51]	2015	USA	1996–2013	HPV+: 56 * (36–81) HPV−: 59 * (37–73)	HPV+ 225/27 HPV− 27/12	291	12.7	OP HPV+: 28 (11.1%) HPV−: 9 (23.1%)
Yao et al. [52]	2011	USA	2000–2006	57 ^§^ (26–90)	224/60	284	15.8	O; OP; HP; L; CUP

Abbreviations: CUP: carcinoma of unknown primary; HP: hypopharyngeal; HPV: human papilloma virus; L: laryngeal; NA: not available; NP: nasopharyngeal; O: oral; OligoM: oligo-metastasis; OP: oropharyngeal; PolyM: poly-metastasis; * presented as mean ± SD; ^§^ presented as median (range).

**Table 2:** Tumor features and TNM data of the included studies.

Author	Year	Primary SCC Site	T-Stage (No. Cases (%))	N-Stage (No. Cases (%))	Multiple Metastasis (No. Cases (%))
Abbas et al. [19]	2018	O	NA	^a^ N0 54 (62%); N1 15 (17%); N2a 2 (2%); N2b 15 (17%); N2c 2 (2%)	1 (16.7%)
Aires et al. [20]	2017	O	T1–2 6 (23%); T3–4 20 (76.9%)	N− 4(15.4%); N+ 22 (84.6%)	6 (23.1%)
Al-Othman et al. [21]	2003	OP	^a^ T1–T2 444 (51%); T3 337 (39%); T4 92 (10%)	^a^ N0 343 (39%); N1 118 (14%); N2 335 (38%); N3 77 (9%)	24 (20%)
Albergotti et al. [22]	2018	OP HPV+	PolyM+: Tx 1 (4.17%); T1 5 (20.83%); T2 12 (50%); T3 3 (12.5%); T4 5 (19.23%) OligoM+: T1 1 (7.14%); T2 7 (50%); T3 2 (14.29%); T4 2 (16.67%)	PolyM+: N1 2 (8.33%); N2a 1 (4.17%); N2b 13 (50%); N2c 5 (19.23%); N3 5 (20.83%) OligoM+: N1 3 (21.43%); N2a 1 (7.14); N2b 6 (50%); N2c 1 (8.33%); N3 1 (7.14%)	26 (68.4%)
Barros-Silva et al. [23]	2020	O; OP	T1–2 7 (31.8%); T3–4 15 (68.2%)	N0 8 (36.4%); N+ 14 (63.6%)	NA
Berzenji et al. [24]	2021	O; OP; HP; L; NP; sinus; CUP	NA	NA	OligoM+ 115 (35.5%) Explosive 64 (19.8%) Explosive/ disseminating 145 (44.8%)
Caballero et al. [25]	2008	HP; L	T1–2 9 (37.5%); T3–4 15 (62.5%)	N0–1 8 (33.3%); N2–3 16 (66.6%)	7 (29.1%)
Calhoun et al. [26]	1994	O; OP; HP; L	^b^ T1 7%; T2 11%; T3 13%; T4 18%	^b^ N0 8%; N1 14%; N2 15%; N3 23%	17 (20%)
Chiesa-Estomba et al. [27]	2021	O; OP; HP; L; NP	T1–2 3 3 (10.3%); T3–4 26 (89.6%)	N0 7 (24.1%); N+ 22 (75.8%)	NA
Coca-Pelaz et al. [28]	2012	O; OP; HP; L	T1–2 13 (21.6%); T3–4 47 (78.3%)	N0–1 13 (21.6%); N2–3 47 (78.3%)	24 (40%)
Dragovic et al. [29]	2013	O; OP; HP; L	^a^ T1 56 (10%); T2 161 (29%); T3 169 (30%); T4 174 (31%)	^a^ N0 183 (33%); N1 77 (14%); N2 247 (44%); N3 53 (10%)	10 (18%)
Duprez et al. [30]	2017	O; OP; HP; L; CUP	T1–2 49 (34.7%); T3–4 75 (53.1%)	N0–1 35 (24.8%); N2–3 104 (73.7%)	NA
Fleming et al. [31]	2021	OP HPV+	NA	NA	57 (69.5%)
Gaffney et al. [32]	2023	OP HPV+	T1–T2 20 (38%); T3–T4 32 (62%)	N0 1 (2%); N1 36 (69%); N2 14 (27%); N3 1 (2%)	20 (38.5%)
Garavello et al. [33]	2006	O; OP; HP; L	T1–2 44 (24.3%); T3–4 137 (75.7%)	N0–1 92 (50.8%); N2–3 89 (49.2%)	55 (30.4%)
Gumusay et al. [34]	2015	O; OP; HP; L; NP	^a^ T1–2 138 (47); T3 81 (28); T4 65 (22)	^a^ N0 112 (38); N1 54 (19); N2 108 (37); N3 12 (4)	8 (29.6%)
Hasegawa et al. [35]	2015	O	T1–2 16 (53.3%); T3–4 14 (46.7%)	N0–1 16 (53.3%); N2–3 14 (46.7%)	3 (10%)
Hauswald et al. [36]	2011	OP; HP	^a^ T2 9 (7%); T3 24 (19%); T4 94 (74%); Tx 3 (2%)	N0–1 4 (8.8%); N2–3 41 (91.1%)	16 (36%)
Huang et al. [37]	2013	OP	HPV+: T1–2 25 (46.3%) T3–4 29 (53.7%) HPV−: T1–2 8 (32%) T3–4 17 (68%)	HPV+: N0–1 6 (11.1%) N2-3 48 (88.8%) HPV−: N0–1 6 (24%) N2–3 19 (76%)	HPV+ 18/54 (33%) HPV− 0/25 (0%)
Kang et al. [38]	2016	O; OP; HP; L; NP; sinuses	T1 19 (19%); T2 24 (25%); T3 20 (20%); T4 35 (36%)	N0 25 (26%); N1 14 (14%); N2 55 (56%); N3 4 (4%)	30 (30.6%)
Kowalski et al. [39]	2005	O; OP	^a^ T1 161 (7%); T2 475 (20%); T3 527 (23%); T4 1164 (50%)	^a^ N0 936 (40%); N1 535 (23%); N2a/b 450 (19%); N2c 258 (11%); N3 130 (6%); NX 18 (1%)	7 (7.8%)
Krstevska et al. [40]	2010	O; OP; HP; L	^a^ T1 6 (3%); T2 72 (36%); T3 87 (43%); T4 36 (18%)	^a^ N0 120 (60%); N1 43 (21%); N2 35 (17%); N3 3 (2%)	4 (15.4%)
Lee et al. [41]	2012	O; OP; HP; L	T1–2 13 (36.1%); T3–4 23 (63.8%)	N0–1 15 (41.6%); N2–3 21 (58.3%)	12 (33.3%)
León et al. [42]	2000	O; OP; HP; L; NP	^c^ T1–2 16 (25%); T3–4 48 (75%)	^c^ N0–1 35 (54.7%); N2–3 29 (45.3%)	20 (11.2%)
Lim J-Y et al. [43]	2010	O; OP; HP; L	cT1–2 23 (5.5%); cT3–4 21 (8.9%) pT1–2 17 (5.8%); pT3–4 27 (10.8%)	cN− 11 (3.1%); cN+ 33 (12%) pN− 9 (5.8%); pN+ 35 (11.5%)	7 (16%) * * among the DM with LRC group (44)
Lim Y-C et al. [44]	2007	O; OP	cT1–2 8 (57.1%); cT3–4 6 (42.8%) pT1–2 7 (50%); pT3–4 7 (50%)	cN- 2 (14.3%); cN+ 12 (85.7%) pN- 1 (7.1%); pN+ 12 (85.7%)	1 (7.1%)
Mattioli et al. [45]	2022	OP	p16+ 35 (61.4%): cT1–2 8 (22.8%); cT3–4 27 (77.1%) p16− 21 (36.8%): cT1–2 5 (23.8%); cT3–4 16 (76.2%)	p16+ 35 (61.4%): cN0–1 18 (51.4%); cN2–3 17 (48.5%) p16− 21 (36.8%): cN0–1 5 (23.8%); cN2–3 16 (76.1%)	4 (7%)
McBride et al. [46]	2013	OP	^a^ T1–T3 (75%); T4 (25%)	^a^ N0-2b (50%); N2c-3 (50%)	14 (56%)
Merino et al. [9]	1977	O; OP; HP; L; sinus	T1–2 7.8%; T3–4 14%	N0–1 6.3%; N2–3 24%	50 (9.1%)
Osaki et al. [47]	2000	O; OP; sinus	T1–2 19 (45.3%); T3–4 23 (54.7%)	N− 17 (40.5%); N+ 25 (59.5%)	5 (11.9%)
Shingaki et al. [48]	1996	O; OP; sinus	T1–2 10 (47.6%); T3–4 11 (52.3%)	N0–1 12 (57.1%); N2 9 (42.8%)	7 (33.3%)
Shintani et al. [49]	1995	O (tongue)	T1–2 14 (63.6%); T3–4 8 (36.6%)	N0 3; N+ 15	3 (13.6%)
Tomioka et al. [50]	2021	O	NA	N− 5 (13.9%); N+ 31 (86.1%)	NA
Trosman et al. [51]	2015	OP HPV+: 28 (11.1%) HPV-: 9 (23.1%)	^a^ HPV+: T1-2 151(60%); T3–4 101 (40%) HPV−: T1-2 6 (15%); T3–4 33 (85%)	^a^HPV+: N0 6 (3%); N1-2a 51 (20%); N2b-3 195 (77%) HPV−: N0 4 (10%); N1-2a 11 (28%); N2b-3 24 (62%)	HPV+: 9 (32%) HPV−: 1 (11%)
Yao et al. [52]	2011	O; OP; HP; L; CUP	NA	NA	7 (15.6%)

Index: ^a^ TNM of all patients (including DM patients); ^b^ raw data not extractable; ^c^ TNM of DM patients with locoregional failure. Abbreviations: CUP: carcinoma of unknown primary; DM: distant metastasis; HP: hypopharyngeal; HPV: human papilloma virus; L: laryngeal; NA: not available; NP: nasopharyngeal; O: oral; OP: oropharyngeal; PolyM: poly-metastasis; OligoM: oligo-metastasis.

**Table 3:** Metastasis features of the included studies.

Author	Year	Primary SCC Site	Metastasis Sites (No. Cases (%))	Locoregional Recurrence (No. Cases (%))	Time to Metastasis Diagnosis (months)	Treatment (No. Cases (%))	Survival
Abbas et al. [19]	2018	O	^a^ Brain 3 (50%); Lg 2 (33%); brain + Lg 1 (17%)	23 (26.1%) ^b^	NA	S 11 (13%); ND 78 (88.6%); S + RT 73 (83%); S + CTRT 4 (5%)	OS 77.3%
Aires et al. [20]	2017	O	^c^ Lg (89%); B (15%); axillary LN (8%); Lv (8%); pleura (7%)	13 (50%)	12 * (2–40)	S 95 (35%); S + RT 108 (39%); S + CTRT 71 (26%)	NA
Al-Othman et al. [21]	2003	OP	^a^ Lg 56 (46%); B 42 (34%); other 24 (20%)	NA	12§	RT 551 (63%) RT + ND 322 (37%)	5y DSS 70% 5y DFS 86%
Albergotti et al. [22]	2018	OP HPV+	^a^ PolyM+: Lg 18 (69%); other sites 8 (31%) OligoM+: Lg 11 (92%)	PolyM+: 8 (30.8%) OligoM+: 5 (41.7%)	PolyM+:17 * OligoM+:23 *	^e^ PolyM+: CT 13; SRS or BCT 2; SRS or CTRT 3; MSC + adj CT 1 OligoM+: MSC 6; NeoadjCT + MSC1; CT 3; MSC + CRT1; MSC + adj CT 1	PolyM+: * OS * 2y 15%; OligoM+: OS * 2.6y 58%; * DFS 20.7mos
Barros-Silva et al. [23]	2020	O; OP	^a^ Lg 9 (41%); brain 6 (27%); B 5 (23%); larynx 1 (4%); Lv 1 (4%)	NA	NA	None 55 (14%); S 48 (12%); S + RT 97 (24%); S + RT + CT 21 (5%); RT 100 (25%); RT + CT 83 (21%)	15y OS 47%
Berzenji et al. [24]	2021	O; OP; HP; L; NP; sinus; CUP	^c^ Lg 187 (58%); mediastinal LN 164 (51%); skin 66 (20%); B 62 (19%); Lv 45 (14%); brain 7 (2%); adrenal glands 7 (2%); parotid glands 5 (1%); spleen 5 (1%); kidneys 5 (1%); pancreas 1 (0.3%); other 52 (16.0%)	Local: 27 (8.3%) Regional: 48 (14.8%) LR: 22 (6.8%)	13.8 * ± 12.3	^e^ None 240 (74%); S or RT 65 (20%); CT 13 (4%); CTRT or CT + S 6 (2%)	^§^ DSS 3.2 mos; * DSS 6.3 mos
Caballero et al. [25]	2008	HP; L	^a^ Lg 10 (70.8%); pleura 2 (11.7%); B 4 (23.5%); Lv 1 (5.8%); multiple DM 7 (29.1%)	Local: 9 (9.5%) Regional: 4 (11.8%)	19.1 * (1–41)	TLM	OS 2.8 mos
Calhoun et al. [26]	1994	O; OP; HP; L	^c^ Lg 69 (83%); B 26 (31%); Lv 5 (6%); brain 2 (2%)	24 (28.9%)	11.7*	^e^ S 15 (18%); RT 37 (44%); S + RT 28 (33.7%); none 3 (4%)	* time from diagnosis of DM to death 4.3 mos
Chiesa-Estomba et al. [27]	2021	O; OP; HP; L; NP	^a^ Lg 25 (86.2%); Lv 3 (10.3%); skin 1 (3.4%)	NA	NA	NA	NA
Coca-Pelaz et al. [28]	2012	O; OP; HP; L	^a^ Lg 33 (55%); B 2 (3%); Lv 1 (2%); multiple DM 24 (40%)	16 (26.6%)	NA	S 443 (100%); ND 392 (88%); Adj RT 216 (49%)	NA
Dragovic et al. [29]	2013	O; OP; HP; L	^a^ Lg 28 (50%); multiple DM 10 (18%); B 6 (11%); skin 4 (7%); mediastinum 2 (3%); Lv 2 (3%); brain 2 (3%); gastrostomy site 1 (2%); pancreas 1 (2%)	27 (48.2%)	16 *	Neoadj CT 62 (11%); RT 205 (37%); CTRT 355 (63%)	3y OS 59%; 3y DMFS 87%; ^§^ OS after DM 5 mos
Duprez et al. [30]	2017	O; OP; HP; L; CUP	^c^ Lg 110 (78%); B 42 (29.7%); Lv 24 (17%); LN outside neck 28 (19.8%); skin 16 (11.3%); pleura 13 (9.2%); adrenal gland 2 (1.4%); soft tissues 2 (1.4%); pancreas 2 (1.4%); brain 1 (0.7%); omentum 1 (0.7%); spleen 1 (0.7%)	64 (45%)	70% ≤ 1 y; 89% ≤ 2 y	S 227 (22%); ND 361 (35%); CT 318 (31%); RT (100%)	1y DMFS 67% 2y DMFS 55% 5y DMFS 41% 10y DMFS 29%
Fleming et al. [31]	2021	OP HPV+	^c^ Lg 61 (74%); B 23 (28%); Lv 10 (12%); axillary LN 5 (6%); brain 3 (4%)	26 (35.1%)	NA	NA	^§^ OS after DM 14.6 mos
Gaffney et al. [32]	2023	OP HPV+	^c^ Lg 33 (64%); thoracic nodes 18 (37%); B 14 (27%); visceral 12 (23%); brain 2 (4%); skin 1 (2%)	NA	15.1 ^§^ (2.6–63)	^e^ Ind CT 10 (19%); RT 17 (33%); CTRT 35 (67%)	NA
Garavello et al. [33]	2006	O; OP; HP; L	^c^ Lg 101 (56%); B 18 (10%); Lv 7 (4%)	72 (5.4%)	NA	NA	NA
Gumusay et al. [34]	2015	O; OP; HP; L; NP	^a^ Lg 13 (48.2%); multiple DM 8 (29.6%); B 5 (14.8%); Brain 2 (7.4%)	33 (11.3%) ^b^	NA	NA	5y DMFS 87%
Hasegawa et al. [35]	2015	O	^a^ Lg 17 (38%); multiple DM 16 (36%); B 5 (11%); Lv 4 (9%); brain 2 (4%); skin 1 (2%)	17 (58.7%)	NA	S 174 (39%); ND 277; Adj RT 76 (17%); Adj CT 38 (8%)	5y OS DM with LRF 0% 5y OS DM with LRC 26.9%
Hauswald et al. [36]	2011	OP; HP	^a^ Lg (38%); multiple DM (36%); B (11%); Lv (9%); brain (4%); skin (2%)	28 (62%)	8 ^§^	CTRT 127 (100%)	1y OS DM 72%
Huang et al. [37]	2013	OP	^a^ HPV+: Lg 42 (78%); skin 12 (22%); brain 8 (15%); abdominal LN 8 (15%); muscle 3 (6%); pancreas 2 (3.7%); axilla 2 (4%); spleen 1 (2%); kidney 1 (2%); pericardial LN 1 (2%) HPV−: Lg 22 (88%); Lv 4 (16%); B 3 (12%)	HPV+: Local 3 (5.5%) Regional 6 (11.1%) LR 6 (11.1%) HPV−: Local 4 (16%) Regional 5 (20%)	NA	^e^ HPV +: Palliative CT 3/54 S 6/54 Palliative RT 2/54 HPV−: Palliative CT 1/25 on lung M+	2y DSS DM HPV+ 1% 2y DSS DM HPV− 4%
Kang et al. [38]	2016	O; OP; HP; L; NP; Sinuses	^a^ Lg 74 (76%); B 23 (24%); mediastinum 3 (3%); skin 3 (3%); Lv 2 (2%); brain 2 (2%); pleura 2 (2%); heart 1 (1%)	Local 8 (8%) Regional 27 (28%) LR 14 (14%)	15 ^§^ (1–87)	S 272 (35%); S + RT ± CT 268 (34%); RT 93 (12%); CTRT 141 (18%)	2y OS DM 36.7% 2y OS DM and LRF 2.8% 2y OS single M+ 26.2% 2y OS polyM+ 7.1%
Kowalski et al. [39]	2005	O; OP	^a^ Lg 45 (2%); B 28 (1%); Lv 2 (0.1%); brain 2 (0.1%); soft tissues 2 (0.1%); peritoneum 1 (0.04%); mediastinum 1 (0.04%); axillary LN 1 (0.04%); multiple DM 7	Local 16 (17.9%) Regional 19 (27.9%)	31.5% in 6	S 637 (27%); RT 1147 (49%); S + RT 543 (23%)	5y DMFS 93.3%
Krstevska et al. [40]	2010	O; OP; HP; L	^a^ Lg 18 (69%); Lv 3 (11%); B 1 (4%); multiple DM 4 (15%)	18 (69.2%)	16.5 * ± 7.5 (5–35)	S + RT 117 (58%); RT 84 (50%)	5y DMFS 84.8%
Lee et al. [41]	2012	O; OP; HP; L	^c^ Lg (81%); B (39%); Lv (11%)	16 (20.5%)	12 * (2–38)	S 308; RT 42; Neoadj CT 28; CTRT 26	2y OS singleM+ 25.7% 2y OS multipleM+ 0%
León et al. [42]	2000	O; OP; HP; L; NP	^d^ Lg 33 (52%); B 8 (12%); Lv 3 (5%); multiple DM 20 (31%)	115 (64.2%)	12 *	Ind CT 408	5y DMFS 94%
Lim J-Y et al. [43]	2010	O; OP; HP; L	^d^ Lg (64%); B (9%); Lv (3%); skin (1%); axilla (1%); cavernous sinus (1%)	31 (41.3%)	13 * (2–70)	S 296 (47%); S + RT 335 (53%) U-ND 391 (62%); B-ND 152 (24%)	5y DMFS 87.5%
Lim Y-C et al. [44]	2007	O; OP	^a^ Lg 5 (36%); Lv 3 (21%); B 2 (14%)	3 (21.4%)	10 ^§^	ND 212 (92%); Adj RT 130 (57%)	^§^ OS after DM 5 mos
Mattioli et al. [45]	2022	OP	^a^ Lg 37 (65%); other 20 (35%)	25 (43.8%)	NA	S 33 (8%); RT 78 (19%); CTRT 215 (53%); S + RT 33 (8%); S + CTRT 44 (11%); None 5 (1%)	5y OS DM 37%; 5y OS no-DM 76%; § OS after DM 34 mos
McBride et al. [46]	2013	OP	^a^ Lg 22 (64%); Lv 4 (12%); B 4 (12%); LN outside neck 4 (12%)	4 (16%)	^§^ 7.9 (1.6–25.4)	^e^ CTRT 23; Ind CT + RT 4; S + RT 1	^§^ OS after DM 18.3 mos; 1y OS after DM 72%; 2y OS after DM 41%
Merino et al. [9]	1977	O; OP; HP; L; Sinus	^a^ OligoM+: Lg 284 (52%); B 111 (20%); Lv 33 (6%); other 52 (9.5%) mediastinum (3%) PolyM+: Lg + B 18 (3.3%); other 32 (5.9%)	^b^ 570 (11.3%)	4% in 9 80% in 24	RT 2819; S 1686; Neo-adj RT 161; Adj RT 353	NA
Osaki et al. [47]	2000	O; OP; Sinus	^a^ Lg 30 (71.4%); Lg + other 5 (11.9%); other (Lg excluded) 7 (16.6%)	25 (59.5%)	NA	S 179; CRT + IMT 327; CTRT 133	NA
Shingaki et al. [48]	1996	O; OP; Sinus	^c^ Lg 18 (85.7%); B 5 (23.8%); skin 5 (23.8%); brain 3 (14.2%); Lv 1 (4.7%)	NA	14 * (5–35)	Elective ND 44 Therapeutic ND 59	NA
Shintani et al. [49]	1995	O (Tongue)	^c^ Lg 13 (50%); B 5 (19%); skin 3 (11%); mediastinum 2 (8%); Lv 1 (4%); brain 1 (4%); pleura 1 (4%)	NA	(6–36)	ND 93 (42%)	NA
Tomioka et al. [50]	2021	O	^c^ Lg 31 (86.1%); B 14 (38.9%); Lv 3 (8.3%); mediastinum 2 (5.5%); adrenal gland 1 (2.7%); cerebellum 1 (2.7%); spleen 1 (2.7%)	31 (89.1%)	21.3 * (2–94)	S 775; BCT 112	NA
Trosman et al. [51]	2015	OP HPV+: 28 (11.1%) HPV−: 9 (23.1%)	^c^ HPV+: Lg 23 (83%); B 12 (21.1%); Lv 7 (12.3%); intra-abdominal lymph nodes 3 (5.3%); axillary lymph nodes 2 (3.5%); brain 2 (3.5%); kidney 2 (3.5%); muscle 2 (3.5%); skin 2 (3.5%); pericardium 1 (1.8%); peritoneum 1 (1.8%); HPV−: 9; Lg 7 (78%); B 2 (16.7%); brain 2 (16.7%) Lv 1 (8.3%)	^b^ HPV+: Local 14 (5.5%) Regional 12 (4.7%) HPV−: Local 8 (20.5%) Regional 4 (10.2%)	NA	CT: 252 HPV+; 39 HPV− RT: 252 HPV+; 39 HPV- Salvage ND: 2 HPV+; 1 HPV	HPV+: 3y OS 90%; ^§^ OS 25.6 mos; HPV−: 3y OS 62%; ^§^ OS 11.1 mos
Yao et al. [52]	2011	O; OP; HP; L; CUP	^c^ Lg 37 (82.2%); Lv 7 (4.4%); B 5 (6.6%); axilla/mediastinum 3 (6.6%); Lg + B 2 (4.4%); Lg + Lv 5 (11.1%)	6 (13.3%)	6.6 * (1.0–39.97)	CTRT 136; RT 50; S + RT 85; S + CTRT 13	3y OS 68.9%; 3y DMFS 84.1%

*Index*: ^a^ percentage of all patients with DM; ^b^ value and percentage including all patients; ^c^ percentage of all DM sites; ^d^ percentage of patients with DM and locoregional control; ^e^ treatment of DM. *Abbreviations* *: mean; ^§^ median; Adj: adjuvant; B: bone; BCT: brachytherapy; B-ND: bilateral nodal dissection; CTRT: chemo-radiotherapy; DFS: disease-free survival; PolyM: poly-metastasis; DM: distant metastasis; DMFS: distant metastasis-free survival; DSS: disease-specific survival; IMT: immunotherapy; Ind CT: induction chemotherapy; HP: hypopharyngeal; HPV: human papilloma virus; L: laryngeal; NA: not available; NP: nasopharyngeal; Lg: lung; LN: lymph node; LR: locoregional; Lv: liver; Mos: months; MSC: metastatectomy; ND: nodal dissection; Neoadj: neo-adjuvant; OS: overall survival; RT: radiotherapy; S: surgery; SRS: stereotactic radio-surgery; TLM: transoral laser microsurgery; U-ND: unilateral nodal dissection; y: year.

**Table 4:** Univariate meta regression results.

Variable		Coefficient (95% CI)	*p*-Value
Primary tumor site	Oral cavity	1 (reference)	-
	Oropharynx NOS	4.66 (−0.91; 10.22)	0.101
	Oropharynx HPV+	5.54 (0.16; 10.92)	**0.044**
	Mixed series	2.41 (−1.76; 6.58)	0.257
Geographic area	Europe	1 (reference)	-
	North America	0.25 (−3.00; 3.49)	0.881
	Asia	−3.47 (−6.73; −0.22)	**0.037**
	Other areas	−3.98 (−9.06; 1.10)	0.124
Publication year		−0.05 (−0.20; 0.09)	0.484

Abbreviations: CI = confident interval; HPV = human papilloma virus; NOS = not otherwise specified.

## Data Availability

The data presented in this study are available on request from the corresponding author.
